# The recovery status from delayed graft function can predict long-term outcome after deceased donor kidney transplantation

**DOI:** 10.1038/s41598-017-14154-w

**Published:** 2017-10-20

**Authors:** Juhan Lee, Seung Hwan Song, Jee Youn Lee, Deok Gie Kim, Jae Geun Lee, Beom Seok Kim, Myoung Soo Kim, Kyu Ha Huh

**Affiliations:** 1 Department of Transplantation Surgery, Severance Hospital, Yonsei University Health System, Seoul, Republic of Korea; 2 Department of Nephrology, Severance Hospital, Yonsei University Health System, Seoul, Republic of Korea

## Abstract

The effect of delayed graft function (DGF) recovery on long-term graft outcome is unclear. The aim of this study was to examine the association of DGF recovery status with long-term outcome. We analyzed 385 recipients who underwent single kidney transplantation from brain-dead donors between 2004 and 2015. Patients were grouped according to renal function at 1 month post-transplantation: control (without DGF); recovered DGF (glomerular filtration rate [GFR] ≥ 30 mL/min/1.73 m^2^); and incompletely recovered DGF group (GFR < 30 mL/min/1.73 m^2^). DGF occurred in 104 of 385 (27%) recipients. Of the DGF patients, 70 recovered from DGF and 34 incompletely recovered from DGF. Death-censored graft survival rates for control, recovered DGF, and incompletely recovered DGF groups were 95.3%, 94.7%, and 80.7%, respectively, at 5 years post-transplantation (*P* = 0.003). Incompletely recovered DGF was an independent risk factor for death-censored graft loss (HR = 3.410, 95%CI, 1.114-10.437). DGF was associated with increased risk for patient death regardless of DGF recovery status. Mean GFRs at 5 years were 65.5 ± 20.8, 62.2 ± 27.0, and 45.8 ± 15.4 mL/min/1.73 m^2^ for control, recovered, and incompletely recovered DGF groups, respectively (*P* < 0.001). Control group and recovered DGF patients had similar renal outcomes. However, DGF was associated with increased risk for patient death regardless of DGF recovery status.

## Introduction

Delayed graft function (DGF) is a common complication after deceased donor kidney transplantation. The association between organ quality and DGF is well established^1^. Notwithstanding that association, there has been increasing use of marginal kidneys due to a critical shortage of organs^2,3^. As a consequence, the incidence of DGF remains high.

In spite of the high incidence, the influence of DGF on long-term outcome is unclear^4^. The lack of uniform DGF definition complicates comparison of research data^5^. In addition, DGF is caused by complex factors with varying severities. The severity of injury can affect the degree of recovery^6^. Moreover, there are recent reports indicating that incomplete recovery from acute kidney injury (AKI) is an important contributor to the progression of chronic kidney disease^7,8^. In the transplantation setting, however, prior studies have focused on the development of DGF *per se*, not only recovery of DGF. For this study, we hypothesised that recovery status might elicit different long-term outcomes.

Some studies have stratified DGF recovery according to dialysis duration^9–13^. However, the decision to discontinue dialysis after DGF is subjective, and currently, there is a lack of consensus for defining DGF recovery. In addition, there are no clearly defined predictive biomarkers for DGF prognosis despite rigorous research^14^. From a practical point of view, renal function after recovery from DGF is probably the best marker for long-term prognosis^15–17^, and glomerular filtration rate (GFR) is accepted as an accurate parameter when assessing renal function^18^. Therefore, we compared transplant outcomes in recipients who exhibited DGF according to DGF recovery status based on GFR.

## Material and Methods

### Patients

A retrospective review of a prospectively collected database of kidney transplants at Severance Hospital revealed 423 adult patients who underwent deceased donor kidney transplantation between 2004 and 2015. Exclusion criteria were simultaneous non-kidney transplantation, en bloc kidney transplantation, and primary non-function. Patients with missing data on donor cause of death, final serum creatinine, donor hypertension, or donor diabetes were excluded. All donors were brain dead.

**Figure 1:**
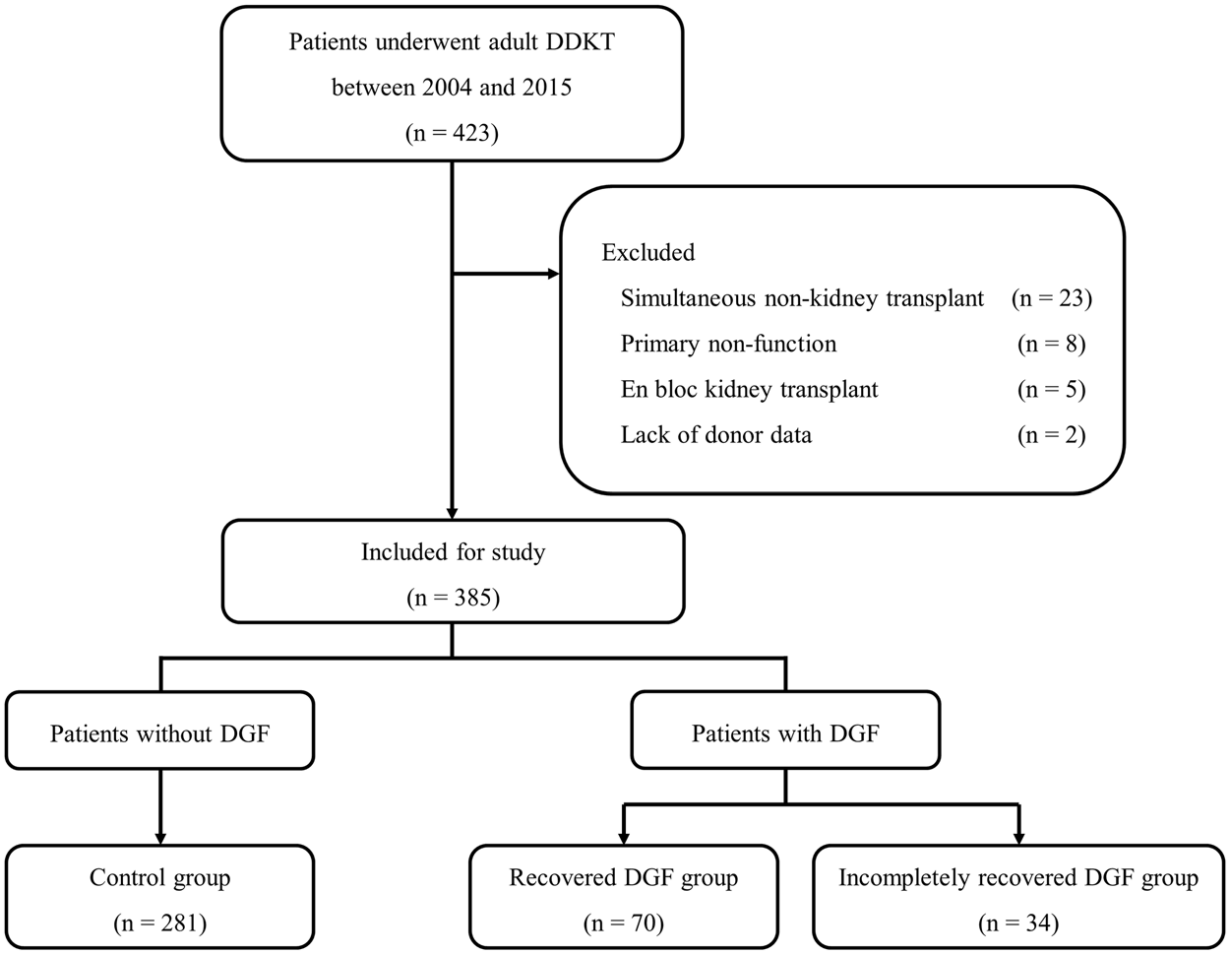
Study flow diagram.

Patients with DGF were grouped into the recovered DGF (GFR ≥ 30 mL/min/1.73 m^2^) or incompletely recovered DGF (GFR < 30 mL/min/1.73 m^2^) groups according to renal function at 1 month post-transplantation. Patients without DGF were assigned to the control group (Fig. 1).

### Definitions

DGF was defined as the need for at least one dialysis session within the first week after kidney transplantation^5^. Expanded criteria donors (ECD) included all donors ≥60 years old and donors ≥50 years old with at least two of the following conditions: history of hypertension; cerebrovascular cause of brain death; or final creatinine >1.5 mg/dL.

### Immunosuppression

All patients received induction therapy with basiliximab or anti-thymocyte globulin (ATG). We selected the induction therapy based on the donor/recipient characteristics and postoperative progress of the patient. Maintenance immunosuppression consisted of calcinurin inhibitors (tacrolimus or cyclosporine), prednisolone, and mycophenolate mofetil (MMF) or mycophenolate sodium (MPS). Initial tacrolimus was administered orally at 0.1 mg/kg twice daily. Subsequent doses were adjusted to maintain a target trough concentration between 3 and 8 ng/mL. Initial oral dose of cyclosporine was 5 mg/kg twice daily, and it was adjusted to achieve a trough level of 100–200 ng/mL. The initial dose of methylprednisolone (500–1000 mg) was gradually reduced to oral prednisolone (5–10 mg/day) during the first 3 weeks after transplantation. Patients received either MMF of 1000–2000 mg/day or MPS of 720–1440 mg/day.

### Outcome assessment

Graft loss was defined as return to long-term dialysis, re-transplant, or death with a functioning graft. Graft loss was defined as return to long-term dialysis, re-transplant, or death with a functioning graft. Graft survival was calculated from the date of transplantation to the date of graft loss or June 30, 2016 (end of follow-up period). In cases of death with a functioning graft, graft survival was censored at the time of death. Patient survival was defined as the length of time from transplantation to the date of death or the date of last follow-up.

All acute rejection (AR) episodes were biopsy proven and classified according to Banff criteria. ARs were classified as acute cellular rejection (ACR) or antibody-mediated rejection (AMR). Biopsies that revealed borderline changes were excluded. For patients with more than one episode of AR, only the first rejection was included in our statistical analysis. Protocol biopsies were not performed during the study period.

Renal function was assessed by using GFR estimated by the modification of diet in renal disease formula (MDRD).

### Statistical analysis

Demographic information was summarised using frequency (percentage), or mean ± standard deviation value, depending on data type. Chi-square tests with Fisher’s exact tests were used to compare categorical variables and one-way Analysis of Variance was used to compare continuous variables. When the data revealed a statistically significant difference, *post hoc* comparisons were performed by applying Bonferroni’s correction for multiple comparisons. Survival and freedom from events were estimated by using the Kaplan-Meier method and were statistically compared by using log-rank tests. Univariate and multivariate analyses were performed by using Cox proportional hazard regression models to determine risk factors for death-censored graft loss, patient death, and incompletely recovered DGF. We included in the multivariate analysis factors significantly differing among the groups in the univariate analyses and also the clinically relevant factors in this respect. Statistical analyses were performed by using SPSS software (version 23.0; SPSS Inc., Chicago, IL, USA), and *P*-values < 0.05 were considered statistically significant.

### Ethics statement

The study procedures were in accordance with the Declaration of Helsinki and were approved by the Institutional Review Board of Severance Hospital (4-2016-1016). Informed consent was waived by the Institutional Review Board because of the study’s retrospective design.

## Results

### Baseline characteristics

A total of 385 patients were included in the study, and DGF occurred in 104 of 385 (27%) recipients. Of the 104 patients with DGF, 70 were in the recovered DGF group and 34 were in the incompletely recovered DGF group. Table 1 summarises the baseline characteristics of the patients stratified by DGF recovery status. The median duration of follow-up was 47 months post-transplantation.

**Table 1:** Baseline characteristics by DGF recovery status.

	No DGF (n = 281)	Recovered DGF (n = 70)	Incompletely recovered DGF (n = 34)	*P*-value
Recipient age at KT	46.89 ± 10.70	46.86 ± 10.58	48.77 ± 10.33	0.617
Male recipient (%)	165 (58.7%)	38 (54.3%)	19 (55.9%)	0.847
Diabetes as the cause of ESRD	31 (11.0%)	10 (14.3%)	5 (14.7%)	0.659
Number of HLA mismatches	2.75 ± 1.55	3.11 ± 1.26	3.01 ± 1.52	0.121
Recipient BMI (kg/m^2^)	22.29 ± 3.41	22.76 ± 3.33	22.19 ± 2.58	0.545
Pretransplant dialysis duration (m)	81.31 ± 47.45	93.79 ± 45.63	86.21 ± 47.12	0.136
Re-transplantation	43 (15.3%)	12 (17.1%)	7 (20.6%)	0.706
Donor age at KT	44.02 ± 13.85	45.66 ± 11.11	49.68 ± 11.68	0.053
Male donor (%)	181 (64.4%)	44 (62.9%)	21 (61.8%)	0.936
Donor BMI (kg/m^2^)	23.08 ± 3.68	22.86 ± 3.37	24.37 ± 4.47	0.122
Mean final serum Cr (mg/dL)	1.21 ± 0.75	1.97 ± 1.41	1.96 ± 1.24	< 0.001*
Donor history of HTN (%)	61 (21.7%)	17 (24.3%)	11 (32.4%)	0.368
Donor history of DM (%)	19 (6.8%)	2 (2.9%)	4 (11.8%)	0.736
Expanded criteria donor (%)	70 (24.9%)	24 (34.3%)	14 (41.2%)	0.060
Cold ischemic time				0.128
<4 hours	90 (32.3%)	22 (31.4%)	9 (25.7%)	
4–8 hours	160 (57.3%)	36 (51.4%)	20 (57.1%)	
8–12 hours	20 (7.2%)	10 (14.3%)	3 (8.6%)	
>12 hours	9 (3.2%)	2 (2.9%)	3 (8.6%)	
Induction therapy (%)				<0.001*
ATG	22 (11.7%)	31 (44.3%)	19 (55.9%)	
Basiliximab	259 (92.2%)	39 (55.7%)	15 (44.1%)	
CNI at discharge (%)				0.419
Tacrolimus	223 (79.4%)	51 (72.9%)	28 (82.4%)	
Cyclosporine	58 (20.6%)	19 (27.1%)	6 (17.6%)	
PRA (%)				0.128
0%	153 (54.4%)	37 (52.9%)	12 (35.3%)	
1 – 20%	44 (15.7%)	6 (8.6%)	6 (17.6%)	
21 – 80%	55 (19.6%)	18 (25.7%)	13 (38.2%)	
>80%	29 (10.3%)	9 (12.9%)	3 (8.8%)	

DGF = delayed graft function, KT = kidney transplantation, DM = diabetes mellitus, ESRD = end stage renal disease, HLA = human leukocyte antigen, BMI = body mass index, Cr = creatinine, HTN = hypertension, ATG = anti-thymocyte globulin, CNI = calcinurin inhibitor, PRA = panel reactive antibodies.

*Post hoc Bonferroni: *P* < 0.05 in all comparisons except recovered DGF vs. incompletely recovered DGF.

There were no significant differences between recipient characteristics among the three groups. The mean level of donor serum creatinine before donation was significantly higher in the DGF group, regardless of recovery status, than in the control group. In the incompletely recovered DGF group, donors were older and ECD were more common compared to those in the recovered DGF group, but the differences were not statistically significant. There was no significant difference in cold ischemic time (CIT) distribution among the groups. Maintenance immunosuppressive regimens were comparable among the groups. Compared to patients without DGF, patients with DGF, regardless of recovery status, were more likely to receive ATG induction. However, the rates of ATG induction were similar between the recovered and incompletely recovered DGF groups.

### Patient and graft survival

**Figure 2:**
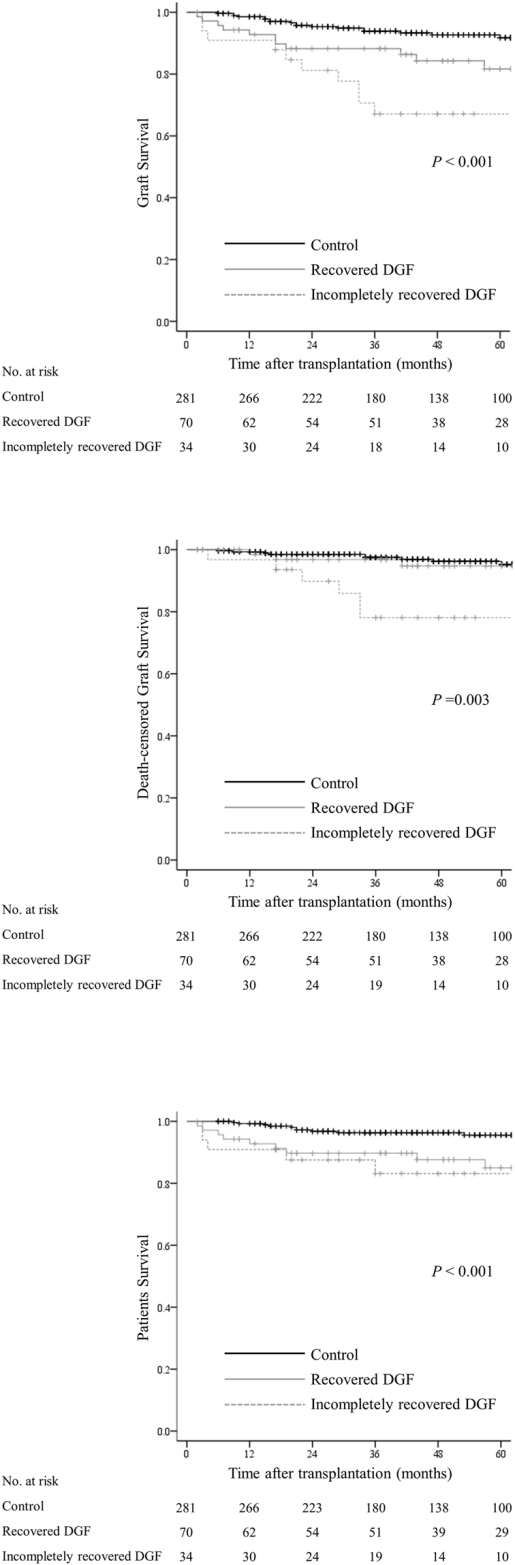
Graft and patient survival according to delayed graft function status. (**a**) Graft survival. (**b**) Death-censored graft survival. (**c**) Patient survival.

**Figure 3:**
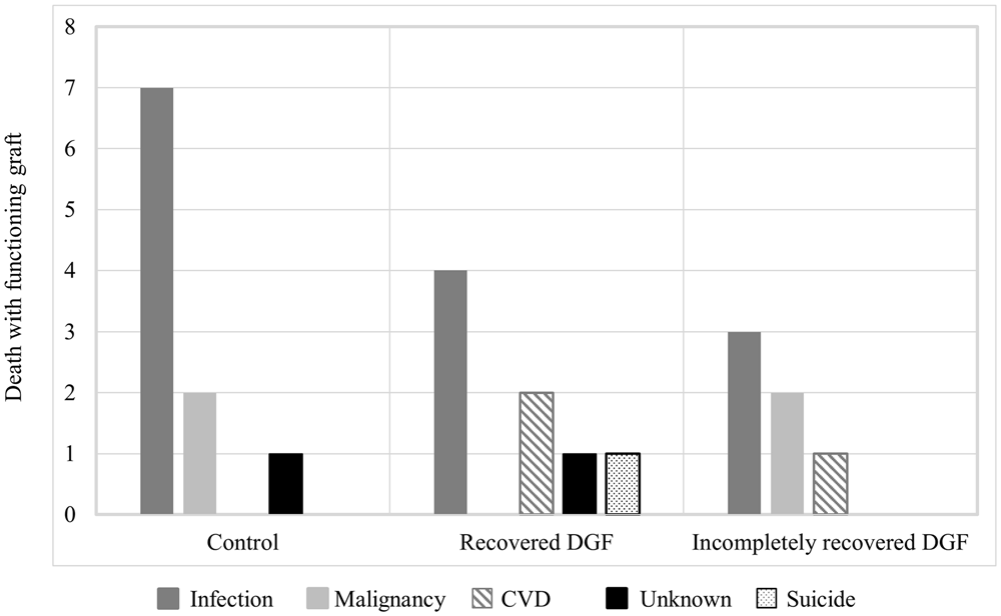
Causes for patient death. CVD: cardiovascular diseases.

Graft and patient survival results are presented in Fig. 2. Five-year all-cause graft losses were 8.2%, 18.3%, and 32.9% for control, recovered DGF, and incompletely recovered DGF groups, respectively (*P* < 0.001). Twenty-four patients died with functioning grafts, accounting for 50% of all graft losses. The predominant cause of death in all groups was infectious diseases (Fig. 3). Death-censored graft survival for control, recovered DGF, and incompletely recovered DGF groups were 95.3%, 94.7%, and 80.7%, respectively, at 5 years post-transplantation (*P* = 0.003). Death-censored graft survival was comparable between the control and recovered DGF groups (*P* = 0.4). Incompletely recovered DGF and acute rejection were independent risk factors for death-censored graft loss (Table 2).

**Table 2:** Risk factor analysis for death-censored graft failure.

	Univariate		Multivariate	
	HR (95% CI)	*P*-value	HR (95% CI)	*P*-value
DGF status				
Recovered	1.549 (0.550, 4.360)	0.407	1.690 (0.574, 4.972)	0.341
Incompletely recovered	4.745 (1.785, 12.619)	0.002	3.410 (1.114, 10.437)	0.032
Acute rejection	4.869 (2.046, 11.583)	<0.001	2.876 (1.064, 7.776)	0.037
HLA mismatches ≥5	1.754 (0.595, 5.173)	0.308		
Recipient age	1.002 (0.962, 1.043)	0.918		
Old donor (≥60 years old)	3.655 (1.442, 9.264)	0.006	1.665 (0.568, 4.882)	0.353
Cold ischemic time	1.061 (0.930, 1.209)	0.379		
Donor creatinine (>1.5 mg/dL)	0.491 (0.183, 1.316)	0.158	0.432 (0.152, 1.225)	0.115
Diabetes as the cause of ESRD	1.476 (0.437, 4.981)	0.531		
Pretransplant dialysis duration (>10 years)	2.200 (0.866, 5.586)	0.097		

DGF = delayed graft function, ESRD = end stage renal disease.

Patient survival did not differ between the recovered and incompletely recovered DGF groups. Five-year patient survival rates were 95.6%, 85.0%, and 83.2%, for control, recovered, and incompletely recovered DGF groups, respectively (*P* < 0.001). Multivariate analysis showed that recipient age, prolonged pretransplant dialysis, and DGF regardless of recovery status were associated with patient death (Table 3).

**Table 3:** Risk factor analysis for patient death.

	Univariate		Multivariate	
	HR (95% CI)	*P*-value	HR (95% CI)	*P*-value
DGF status				
Recovered	3.622 (1.471, 8.919)	0.005	3.029 (1.135, 8.082	0.027
Incompletely recovered	5.482 (1.991, 15.092)	0.001	3.524 (1.189, 10.441)	0.023
Acute rejection	3.014 (1.254, 7.240)	0.014	2.312 (0.896, 5.968)	0.083
HLA mismatches ≥5	1.852 (0.632, 5.428)	0.262		
Recipient age	1.070 (1.026, 1.117)	0.002	1.063 (1.013, 1.116)	0.013
Old donor (≥60 years old)	0.839 (0.198, 3.565)	0.812	0.474 (0.108, 2.080)	0.322
Cold ischemic time	1.073 (0.942, 1.223)	0.289		
Donor creatinine (>1.5 mg/dL)	1.102 (0.487, 2.495)	0.816	0.681 (0.276, 1.683)	0.405
Diabetes as the cause of ESRD	1.656 (0.567, 4.837)	0.356		
Pretransplant dialysis duration (>10 years)	4.133 (1.834, 9.314)	0.001	2.989 (1.303, 6.856)	0.010

DGF = delayed graft function, ESRD = end stage renal disease.

### Renal function

**Figure 4:**
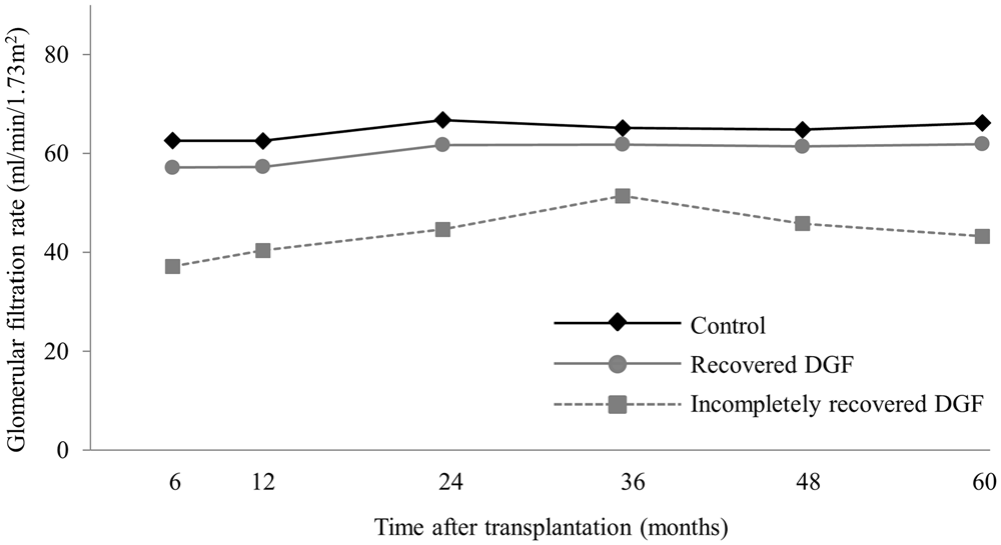
Renal function according to DGF recovery status.

The mean GFRs were consistently lower in the incompletely recovered DGF group than in the control and recovered DGF groups throughout the follow-up period (Fig. 4). Compared to the incompletely recovered DGF group, graft function in the recovered DGF group recovered well and remained stable. From 6 months on, mean GFRs of the recovered DGF and control groups were similar. Mean GFRs at 5-year post-transplantation were 65.5 ± 20.8, 62.2 ± 27.0, and 45.8 ± 15.4 mL/min/1.73 m^2^ for control, recovered DGF, and incompletely recovered DGF groups, respectively (*P* < 0.001).

### Acute rejection

One-year cumulative probabilities of AR in the control, recovered DGF, and incompletely recovered DGF groups were 10.3%, 17.1%, and 23.5%, respectively (log-rank *P* = 0.02). Among the 49 patients with AR, approximately two-thirds of ARs (33/49, 67.3%) occurred within 4 months of transplantation. The histologic features of AR are summarised in Table 4. In the incompletely recovered DGF group, severe ACRs were more common than those of the control and recovered DGF groups, but the differences were not statistically significant. The incidences of AMR were not significantly different among the three groups.

**Table 4:** Histologic features of acute rejection.

	No DGF (n = 281)	Recovered DGF (n = 70)	Incompletely recovered DGF (n = 34)	*P*-value
Overall acute rejection	29 (10.3%)	12 (17.1%)	8 (23.5%)	0.046*
Acute cellular rejection				0.075
Grade I	14 (5.0%)	3 (4.3%)	2 (5.9%)	
Grade II/III	5 (1.8%)	4 (5.7%)	4 (11.8%)	
Acute antibody-mediated rejection				0.273
Pure antibody-mediated rejection	6 (2.1%)	3 (4.3%)	2 (5.9%)	
Mixed rejection	4 (1.4%)	2 (2.9%)	0	

^*^ *Post hoc* Bonferroni: *P* < 0.05 in comparison between control vs. incompletely recovered DGF.

### Risk factors associated with incompletely recovered DGF

Multivariate analysis showed that CIT, donor final creatinine, and old donor were associated with incompletely recovered DGF (Table 5).

**Table 5:** Risk factor analysis for incompletely recovered DGF.

	Univariate		Multivariate	
	HR (95% CI)	*P*-value	HR (95% CI)	*P*-value
Recipient Age	1.005 (0.984, 1.027)	0.628		
Diabetes as the cause of ESRD	1.268 (0.466, 3.456)	0.642		
HLA mismatches ≥5	1.075 (0.310, 3.730)	0.910		
Retransplantation	1.395 (0.579, 3.363)	0.458		
Donor age <40 years old				
40–60 years old	2.130 (0.843, 5.381)	0.110	2.304 (0.874, 6.071)	0.091
>60 years old	3.305 (0.942, 11.589)	0.062	4.761 (1.215, 18.655)	0.025
Cold ischemic time (per hour)	3.767 (1.144, 12.401)	0.029	1.175 (1.051, 1.313)	0.005
Donor final creatinine (mg/dL)	1.442 (1.108, 1.876)	0.006	1.515 (1.138, 2.017)	0.004
Donor HTN	1.674 (0.782, 3.584)	0.185	1.085 (0.472, 2.494)	0.848
Donor diabetes	2.095 (0.675, 6.504)	0.201		
Pretransplant dialysis duration (>10 years)	1.463 (0.632, 3.385)	0.374		

DGF = delayed graft function, ESRD = end stage renal disease, HTN = hypertension.

## Discussion

While the negative consequences of DGF on clinical outcome have been described in many reports, the impact of DGF recovery status on graft outcome has not been reported^4,19^. Defining DGF recovery based on recent AKI criteria is challenging because baseline renal function has not been fully elucidated^20^. Hence, in this study, we apply threshold values for GFR based on chronic kidney disease stage (i.e., stage 4 [GFR < 30 mL/min/1.73 m^2^]: severely reduced renal function), which has been verified and widely used^21^. Our study results indicate that patients with incompletely recovered DGF (GFR < 30 mL/min/1.73 m2) were associated with inferior renal function and death-censored graft survival. In contrast, recovered DGF (GFR ≥ 30 mL/min/1.73 m2) had death-censored graft survival and renal function similar to those without DGF (controls). DGF was an independent risk factor for patient death, regardless of DGF recovery status. The incidence of AR at 12 months has highest in the incomplete DGF recovery group.

DGF results from ischaemia-reperfusion injury (IRI) to the graft tissue^22^. In a non-transplant setting, renal IRI lead to AKI with varying reversibility^23^. There are recent reports that incomplete recovery of renal function after AKI is a strong risk factor for decreased long-term survival and poor renal survival^7,8^. While DGF can be considered a severe form of AKI, little is known about recovery after DGF and its influence on long term outcomes^24^. In addition, it is difficult to precisely define DGF recovery, consequently, several studies have stratified DGF recovery based on the dialysis duration or serum creatinine^9–13,25^.

Kidney has the ability to repair itself, depending on the severity of the initial damage. Incompletely recovered areas may develop into focal fibrosis^26,27^. In fact, patients with severe AKI have inferior long-term renal outcome compared to that in with mild AKI patients^6^. Similar to the adverse effects of AKI, prolonged DGF, as a reflection of severe injury, can worsen renal outcomes^9–12^. Our results demonstrated that incompletely recovered DGF was associated with inferior renal outcomes in terms of death-censored graft survival and renal function. By contrast, recovered DGF patients had long-term renal outcomes similar to those of the control group. It is conceivable that kidneys with recovered DGF were less severely damaged than kidneys with incompletely recovered DGF.

Reports describing the effects of DGF on patient survival are conflicting. Meta-analysis by Yarlagadda *et al*. revealed no significant association between DGF and mortality^4^. In contrast, other recent studies revealed that DGF is associated with an increased risk for death with functioning graft in both deceased and living donor kidney transplant recipients^28,29^. In addition, DGF is an independent risk factor for cardiovascular disease after kidney transplantation^30^. In the present study, DGF was associated with a greater risk of patient death regardless of DGF recovery status, and cardiovascular disease related deaths occurred only in the DGF group.

IRI cause cell damage through several pathways including cell death, microvascular dysfunction, and activation of immune system^31^. In particular, IRI leading to DGF increase the expression of human leukocyte antigen (HLA) molecules on endothelial cell surfaces, thus increasing the immunogenicity of the allograft^24^. Some past reports show no significant effect of DGF on development of AR^32^; however, recent studies indicate that DGF is an important risk factor for AR, even in the modern era^33^. In this study, AR occurred more frequently in patients with incompletely recovered DGF, which is consistent with previous studies showing an association between prolonged DGF and AR^10,12^.

Prior studies revealed that well-established risk factors for developing DGF, such as donor age, CIT, re-transplantation, and HLA mismatches are associated with prolonged DGF^10,12^. In this study, we found that donor final creatinine, old donor, and CIT were associated with incompletely recovered DGF. However, the severity of IRI is dependent on a complex interplay of pre-transplant injury and subsequent immune responses after reperfusion^34^. Furthermore, clinical factors including medical environment, organ donation rate, and allocation system, differ between countries. Hence, it is difficult to generalize and quantify the interaction of clinical factors with the DGF recovery status.

Cut-off value for DGF recovery is inherently arbitrary. Although this study could not identify the optimal cut-off eGFR value for recovery, our data suggest that DGF recovery status based on GFR at 1 month post-transplantation can predict the long-term outcome. Furthermore, this stratification will have an immediate clinical applicability, since it is identical to the widely used cut-off for chronic kidney disease stage. Although there is no effective treatment for DGF, correct stratification of DGF recovery status in the early postoperative period may contribute to improve post-transplant management. Clinical practices including immunosuppressive regimen, threshold for biopsy, and cardiovascular work-up might be tailored to individual patients based on their DGF recovery status.

There are several limitations in this study. First, it was performed retrospectively at a single institution. Second, we did not measure GFR by inulin clearance but rather used estimated GFR by using MDRD equation. However, GFR measurement by inulin clearance is unsuitable for daily clinical practice.

DGF is a clinical entity caused by complex factors with varying severities. Our results suggest that assessment of renal function based on GFR at 1-month after transplantation can provide useful prognostic information about long-term outcome. Patients with incompletely recovered DGF present inferior renal function and death-censored graft survival at 5-years, compared to patients without DGF and patients with recovered DGF. DGF is associated with a greater risk of patient death, regardless of DGF recovery status.
